# Bromodomain-containing protein 7 (BRD7) as a potential tumor suppressor in hepatocellular carcinoma

**DOI:** 10.18632/oncotarget.7637

**Published:** 2016-02-23

**Authors:** Chang-Long Chen, Ying Wang, Qiu-Zhong Pan, Yan Tang, Qi-Jing Wang, Ke Pan, Li-Xi Huang, Jia He, Jing-Jing Zhao, Shan-Shan Jiang, Xiao-Fei Zhang, Hong-Xia Zhang, Zi-Qi Zhou, De-Sheng Weng, Jian-Chuan Xia

**Affiliations:** ^1^ Collaborative Innovation Center for Cancer Medicine, State Key Laboratory of Oncology in South China, Sun Yat-Sen University Cancer Center, Guangzhou, China; ^2^ Department of Biotherapy, Sun Yat-Sen University Cancer Center, Guangzhou, China; ^3^ Department of Epidemiology and Health Statistics, Guangdong Key Laboratory of Molecular Epidemiology, Guangdong Pharmaceutical University, Guangzhou, China

**Keywords:** BRD7, tumor suppressor, hepatocellular carcinoma, prognosis, tumorigenicity

## Abstract

Bromodomain-containing protein 7 (BRD7) is a subunit of the PBAF complex, which functions as a transcriptional cofactor for the tumor suppressor protein p53. Down-regulation of BRD7 has been demonstrated in multiple types of cancer. This study aimed to investigate BRD7 expression and its tumor suppressive effect in hepatocellular carcinoma (HCC). The expression of BRD7 was examined in clinical specimens of primary HCC and in HCC cell lines through real-time quantitative PCR, western blot and immunohistochemistry. The prognostic value of BRD7 expression and its correlation with the clinicopathological features of HCC patients were statistically analyzed. The effect of BRD7 on the tumorigenicity of HCC was also examined using proliferation and colony-formation assays, cell-cycle assays, migration and cell-invasion assays, and xenograft nude mouse models. BRD7 was down-regulated in tumor tissues and HCC cell lines. BRD7 protein expression was strongly associated with clinical stage and tumor size. Kaplan-Meier survival curves revealed higher survival rates in patients with higher BRD7 expression levels compared to those with lower BRD7 levels. A multivariate analysis indicated that BRD7 expression was an independent prognostic marker. The re-introduction of BRD7 expression significantly inhibited proliferation, colony formation, migration and invasion and led to cell cycle arrest in HCC cells *in vitro*. Furthermore, experiments in mice suggested that BRD7 overexpression suppresses HCC tumorigenicity *in vivo*. In conclusions, our data indicated that BRD7 may serve as a tumor suppressor in HCC and may be a novel molecular target for the treatment of HCC.

## INTRODUCTION

Hepatocellular carcinoma (HCC) is now the sixth most prevalent cancer and the second most common cause of cancer-related deaths worldwide, especially in developing countries [1-3]. Although transarterial chemoembolization (TACE), selective intra-arterial radiotherapy (SIRT) and systemic chemotherapy are used as palliatives in HCC patients, surgery and local ablative therapies remain the only curative treatment options for more advanced disease [4]. Sorafenib, which is the only FDA-approved systemic treatment for HCC, is the current standard of care for patients with advanced HCC [5]. However, many patients see no long-term clinical benefits from these aggressive interventions, and the 5-year survival rate is low due to the high incidence of intrahepatic recurrence and/or distant metastases [6]. Therefore, there is a clear and urgent need to explore novel therapeutic strategies for this deadly disease. Hepatocarcinogenesis is a multifactorial and multistep process in which many oncogenes and tumor suppressor genes are altered, including inactivation of the tumor suppressor gene p53, activation of the MYC oncogene and mutations in other genes [7, 8]. Identifying and investigating the genetic alterations involved in HCC development and progression are crucial to understanding of the mechanisms of hepatocarcinogenesis and to improving the prognosis of HCC patients [9].

Bromodomain-containing protein 7 (BRD7), also known as the bromodomain protein of 75 kDa (BP75), is a member of the bromodomain-containing protein family [10]. BRD7 is a crucial subunit of the PBAF (polybromo-associated BRG1-associated factor) chromatin remodeling complex, which is involved in transcriptional regulation through interactions with acetylated histones in chromatin [11-13]. BRD7 was originally identified as a tumor suppressor that inhibits nasopharyngeal carcinoma (NPC) cell growth by negatively regulating the beta-catenin and ERK pathways [14, 15]. More recent studies also suggest that BRD7 functions as a tumor suppressor gene. For example, BRD7 suppresses tumorigenicity by recruiting chromatin-remodeling complexes to the promoters of target genes, affecting histone acetylation, p53 acetylation and promoter activity [16]. BRD7 was also found to regulate BRCA1-dependent transcription through the recruitment of BRCA1 and Oct-1 to the ESR1 promoter [17]. Moreover, BRD7 directly interacts with p85α to negatively regulate PI3K signaling, which maintains homeostatic cell growth [18]. In addition, various reports have confirmed that BRD7 expression is down-regulated and significantly correlates with clinical outcomes in a broad range of malignant tumors, such as osteosarcoma, prostate cancer, colorectal cancer and epithelial ovarian carcinoma [19-22]. These findings clearly indicate the essential role of BRD7 in tumor suppression and in the prognosis of patients with cancer. To date, however, the potential function of BRD7 in HCC tumorigenesis, as well as its expression and prognostic significance in HCC patients, remains poorly understood.

In the current study, we measured the expression of BRD7 in primary HCC samples *via* real-time quantitative RT-PCR, western blot and immunohistochemistry. We also investigated the correlation between BRD7 expression and clinicopathologic features in HCC patients and estimated the prognostic value of BRD7 expression in patient survival. We also evaluated the functional role of BRD7 in HCC tumor progression by examining colony formation, proliferation, cell cycle, migration and invasion *in vitro*. Furthermore, we used injectable mouse models to examine the role of BRD7 in HCC tumorigenicity.

## RESULTS

### Expression of BRD7 in HCC tissue samples and HCC cell lines

The detection of BRD7 mRNA expression *via* reverse transcription required 1 μg of total RNA, while 30 μg of protein was needed to examine BRD7 protein expression. However, RNA and protein can degrade during storage, and sufficient amounts of RNA and protein could not be isolated from some small samples (cancerous tissues or adjacent noncancerous tissues). Thus, of the 40 paired samples obtained from HCC patients, only 34 and 25 paired samples were suitable to perform real-time PCR and western blot analyses, respectively.

**Figure 1:**
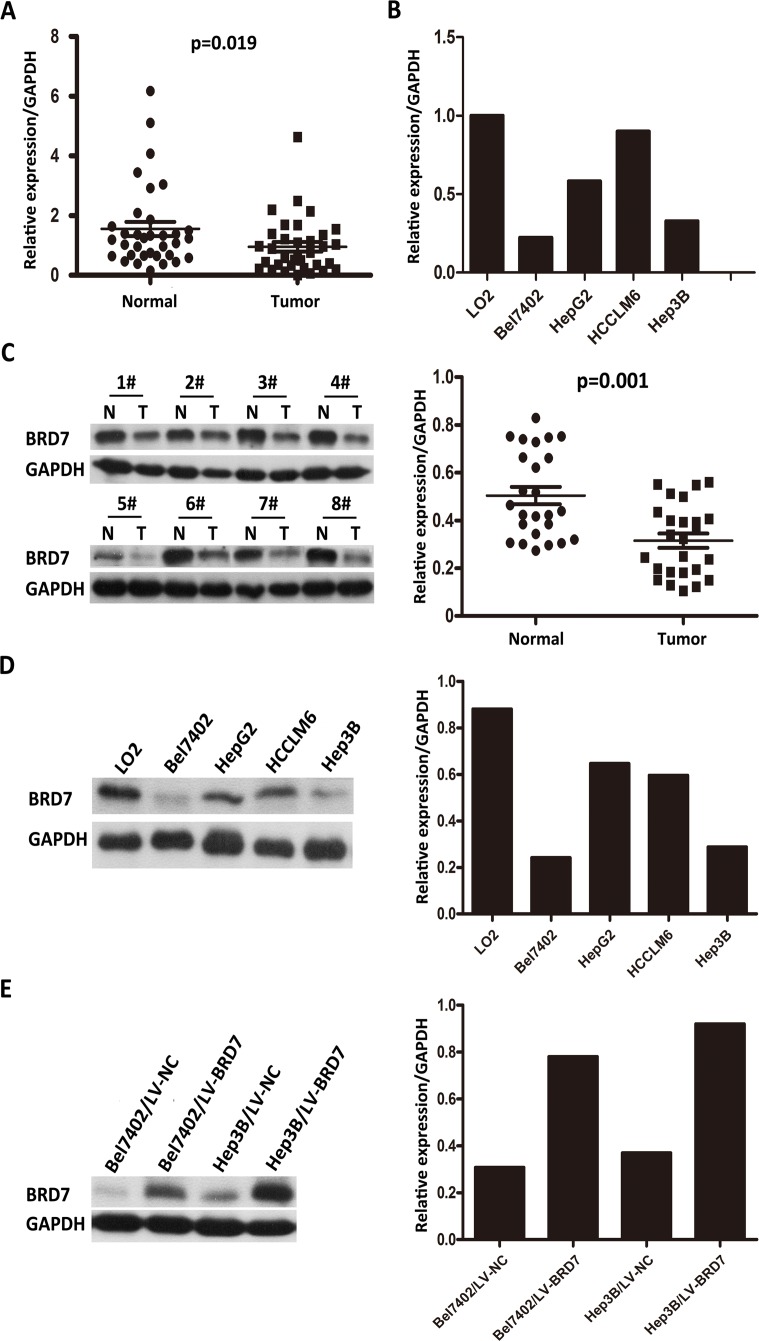
Real-time quantitative PCR and western blot analysis of BRD7 mRNA and protein expression in primary HCC surgical specimens and HCC cell lines **A.** Real-time quantitative PCR analysis of BRD7 mRNA expression in 34 paired tumor tissues and matched adjacent non-tumor tissues (*P* = 0.019). **B.** BRD7 mRNA expression was down-regulated in Bel7402, HepG2, HCCLM6, and Hep3B HCC cells and was particularly low in Bel7402 and Hep3B cells compared with the normal liver cell line LO2. **C.** The left side depicts a western blot of BRD7 protein expression in eight paired HCC tissues (T) and their matched adjacent noncancerous tissues (N); the right side shows that the relative expression of BRD7 protein was significantly lower in the 25 HCC tissues compared with the matched adjacent noncancerous tissues (*P* = 0.001). **D.** BRD7 protein levels were markedly decreased in Bel7402, HepG2, HCCLM6, and Hep3B HCC cells compared with LO2 cells. **E.** BRD7 expression was strikingly higher in LV-BRD7-transfected Bel7402 and Hep3B cells than in cells that were transfected with the LV-Vector.

Real-time quantitative PCR of the 34 paired HCC tissue samples revealed significantly lower BRD7 mRNA expression in tumor tissues compared with adjacent non-tumor tissues (*P* = 0.019; Figure 1A). Moreover, BRD7 transcript levels in the Bel7402, HepG2, Hep3B and HCCLM6 HCC cell lines were decreased compared with those in the normal liver cell line LO2 (Figure 1B). To explore differences in BRD7 expression at the protein level, western blot analyses were performed on the 25 paired tissue samples and on the HCC cell lines. Consistent with the differences in BRD7 mRNA levels, decreased BRD7 protein expression was observed in HCC tumor tissues compared with non-tumor tissues (*P* = 0.001; Figure 1C). Likewise, BRD7 protein levels in Bel7402, HepG2, Hep3B and HCCLM6 cells were also lower than those in LO2 cells (Figure 1D).

### Immunohistochemical analysis of BRD7 expression in HCC tissue samples and its relationship with the clinicopathological features of HCC patients

**Figure 2:**
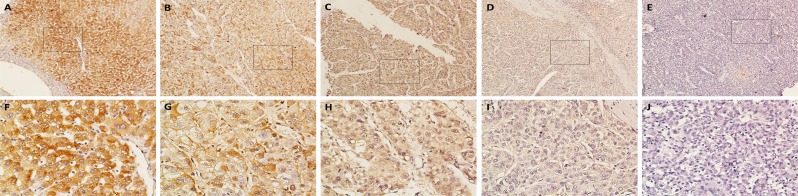
Immunohistochemical analysis of BRD7 protein expression in primary HCC surgical specimens **A.** and **F.** Strongly stained adjacent non-tumor tissues. **B.** and **G.** Well-stained tumor tissues, scored as BRD7+++. **C.** and **H.** Moderately stained tumor tissues, scored as BRD7++. **D.** and **I.** Weakly stained tumor tissues, scored as BRD7+. **E.** and **J.** Negatively stained tumor tissues, scored as BRD7-. **A.**-**E.**, 100× magnification; **F.**-**J.**, 400× magnification).

Immunohistochemical analyses of the 159 paraffin-embedded HCC surgical specimens suggested that BRD7 expression is predominantly localized to the cytoplasm in tumor cells (Figure 2). BRD7 expression was also detected in adjacent non-tumor tissues, which exhibited stronger BRD7 immunoreactivity compared with tumor tissues (Figure 2A and 2F). Overall, 70 of 159 (44.0%) cases exhibited high BRD7 expression (BRD7++ or BRD7+++) in cancerous tissues, whereas 89 (56%) cases exhibited low BRD7 expression (BRD7- or BRD7+) (Table 1). The relationship between BRD7 expression and various clinical characteristics of HCC patients is presented in Table 1. The correlation analysis revealed that decreased BRD7 expression was significantly associated with TNM stage (*P* = 0.002) and tumor size (*P* = 0.008), but not with gender, age, HBsAg, serum AFP, liver cirrhosis, histological differentiation, tumor capsule or tumor number (Table 1).

**Table 1:** Relationship between BRD7 expression and clinical characteristics of patients with HCC

Clinicopathologic variables	Number	BRD7 expression		*P* value
		high	low	
All cases	159	70	89	
Age (years)				0.760
<50	93	40	53	
≥50	66	30	36	
Gender				0.746
Male	129	56	73	
Female	30	14	16	
HBsAg				0.076
Negative	21	13	8	
Positive	138	57	81	
Tumor size(cm)				0.008 *
<5	81	44	37	
≥5	78	26	52	
Tumor number				0.156
Single	136	63	73	
Mutiple	23	7	16	
Liver cirrhosis				0.622
No	30	12	18	
Yes	129	58	71	
Tumor encapsulation				0.354
None	65	33	32	
Complete	52	21	31	
Incomplete	42	16	26	
Serum AFP(ng/ml)				0.810
≤400	96	43	53	
>400	63	27	36	
Histilogical differentiation				0.334
Well	49	20	29	
Moderate	74	37	37	
Poor	36	13	23	
TNM stage				0.002*
I	89	49	40	
II	43	16	27	
III	27	5	22	

HBsAg, Hepatitis B surface antigen; AFP, alpha-fetoprotein.

* Statistically significant (*P* < 0.05).

### Association between BRD7 expression and HCC patient survival

**Figure 3:**
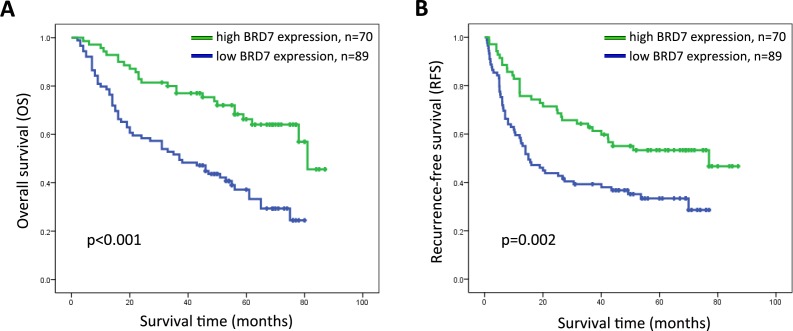
Kaplan-Meier survival curves for HCC patients after hepatectomy according to BRD7 expression **A.** Overall survival (OS) and **B.** recurrence-free survival (RFS) curves are shown. Significantly improved OS and RFS were observed in HCC patients whose tumors exhibited high BRD7 expression (BRD7+++ or BRD7++) *versus* those whose tumors exhibited low BRD7 expression (BRD7+ or BRD7-) (*P* < 0.001 and *P* = 0.002).

The prognostic value of BRD7 expression with respect to HCC patient survival was evaluated *via* Kaplan-Meier survival analysis. The median overall survival (OS) and recurrence-free survival (RFS) rates of patients with high BRD7 expression were 56 and 44 months, respectively, compared with 37 and 15 months for patients with low BRD7 expression. The OS and RFS rates at 5 years were 66.3% and 53.3%, respectively, for patients with high BRD7 expression compared with 37.3% and 33.4% for patients with low BRD7 expression (log-rank test, *P* < 0.001 and *P* = 0.002, respectively). These results indicated that higher BRD7 expression was significantly correlated with improved prognosis in HCC patients (Figure 3). The effect of BRD7 expression on patient survival was further evaluated by univariate and multivariate analyses. In the univariate analysis, BRD7 expression was significantly associated with improved OS and RFS in patients (Table 2 and Table 3). The multivariate analysis further suggested that BRD7 expression is associated with improved OS, although BRD7 expression was not an independent predictor of RFS in HCC patients (Table 2 and Table 3).

**Table 2:** Univariate and multivariate analysis of overall survival in HCC patients

Variables	Univariate analysis			Multivariate analysis		
	HR	95% CI	*p* value	HR	95% CI	*P* value
Age	0.945	0.611-1.463	0.801			
Gender	0.899	0.534-1.515	0.690			
HBsAg	1.161	0.599-2.250	0.658			
Tumor size	1.590	1.032-2.450	0.035*	1.152	0.706-1.880	0.572
Tumor number	2.439	1.442-4.126	0.001*	1.598	0.874-2.920	0.128
Tumor encapsulation	1.278	0.980-1.665	0.070			
TNM stage	1.845	1.398-2.435	<0.0^0^1*	1.419	0.995-2.024	0.054
Liver cirrhosis	0.919	0.539-1.569	0.758			
Histological differeniation	0.979	0.722-1.328	0.891			
AFP	1.131	0.732-1.745	0.579			
BRD7	2.766	1.713-4.465	<0.001*	2.289	1.396-3.753	0.001*

HR, hazard ratio; CI, confidence interval; HBsAg, Hepatitis B surface antigen; AFP, alpha-fetoprotein.

* Statistically significant (*P* < 0.05).

**Table 3:** Univariate and multivariate analysis of recurrence-free survival in HCC patients

Variables	Univariate analysis			Multivariate analysis		
	HR	95% CI	*p* value	HR	95% CI	*P* value
Age	1.256	0.834-1893	0.275			
Gender	0.768	0.468-1.262	0.298			
HBsAg	1.590	0.798-3.168	0.187			
Tumor size	1.695	1.122-2.561	0.012*	1.136	0.698-1.850	0.608
Tumor number	2.770	1.663-4.613	<0.001*	2.207	1.230-3.960	0.008*
Tumor encapsulation	1.354	1.046-1.753	0.021*	1.252	0.961-1.630	0.095
TNM stage	1.817	1.387-2.381	<0.001*	1.425	0.999-2.033	0.051
Liver cirrhosis	1.030	0.608-1.744	0.914			
Histological differeniation	1.159	0.868-1.546	0.317			
AFP	1.540	1.022-2.321	0.039*	1.618	1.056-2.478	0.027*
BRD7	1.934	1.259-2.973	0.003*	1.480	0.942-2.326	0.089

HR, hazard ratio; CI, confidence interval; HBsAg, Hepatitis B surface antigen; AFP, alpha-fetoprotein.

* Statistically significant (*P* < 0.05).

### The role of BRD7 in HCC cell viability

**Figure 4:**
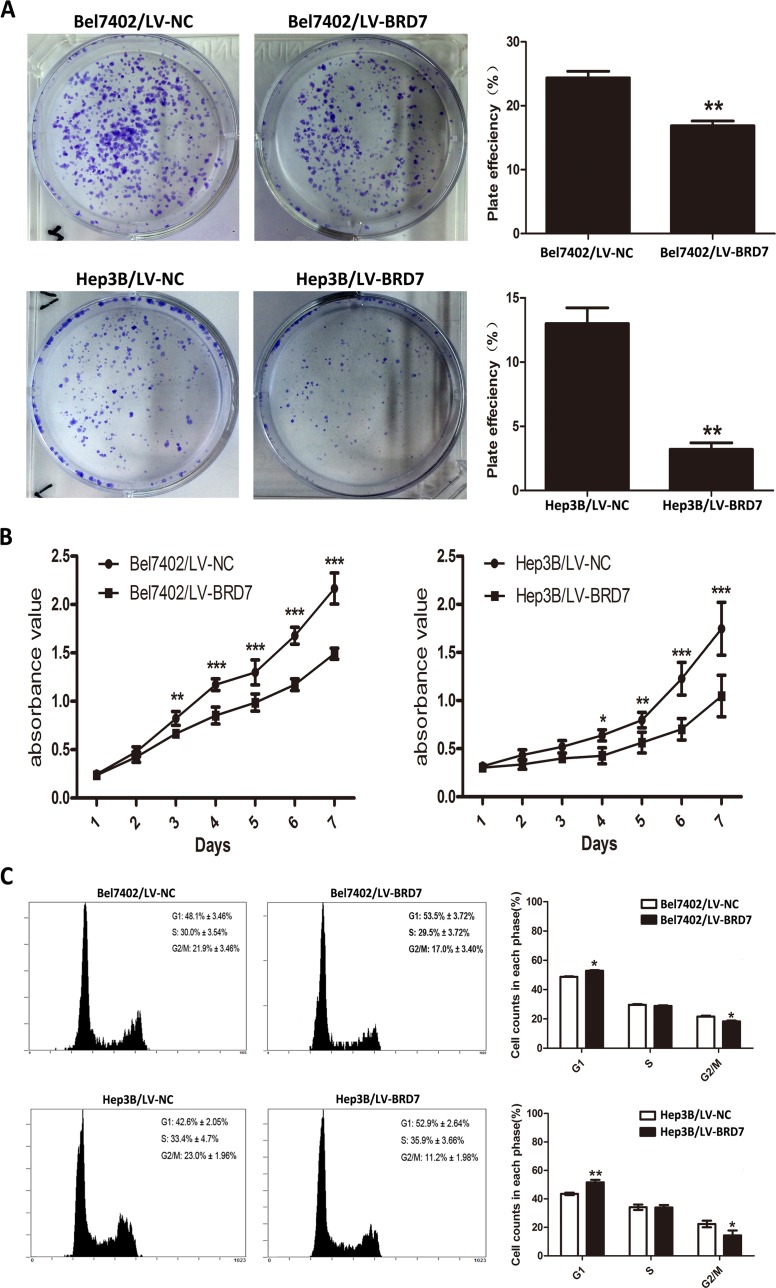
BRD7 overexpression inhibits HCC cell viability **A.** Colony-formation assays indicated that BRD7 decreased the growth rates of Bel7402 and Hep3B cells. **B.** MTS assays showed that BRD7 inhibited the proliferation of Bel7402 and Hep3B cells. **C.** The effect of BRD7 overexpression on the cell cycle. BRD7 overexpression caused G1 arrest and reduced the percentage of Bel7402 and Hep3B cells in G2/M phase. Experiments were performed in triplicate. The mean ± SD of the foci for each group are shown. P-values were calculated using the independent Student's *t*-test. *, *P* < 0.05 *versus* LV-Vector; **, *P* < 0.01 *versus* LV-Vector; ***, *P* < 0.001 *versus* LV-Vector.

Western blot analysis of HCC cell lines showed relatively low BRD7 expression in Bel7402 and Hep3B cells compared with the other cell lines tested (Figure 1D). Thus, we selected Bel7402 and Hep3B cells as the optimal cells for transfection with LV-BRD7 and LV-Vector. The effects of BRD7 overexpression on the transfected cells was confirmed by western blotting (Figure 1E). Colony-formation and cell-proliferation assays were then performed to explore the effect of BRD7 on HCC cell growth. We found that colony formation was significantly reduced in LV-BRD7-transfected Bel7402 and Hep3B cells compared with cells transfected with LV-Vector (Figure 4A). Moreover, the cell-proliferation assay showed that the growth rate of the BRD7-transfected HCC cells was significantly lower than that in the vector-transfected cells (Figure 4B). To elucidate the potential mechanisms by which BRD7 inhibits HCC cell viability, we evaluated the possible effects of BRD7 expression on the cell-cycle distribution by flow cytometric analysis. The results revealed that overexpressing BRD7 in the HCC cells led to G0/G1 phase arrest and reduced the percentage of cells in G2/M phase (Figure 4C). Together with the results of the correlation analysis, which revealed a significant association between decreased BRD7 expression and tumor size, these findings indicated that BRD7 plays key roles in preventing the growth of tumor cells during HCC development.

### BRD7 inhibits HCC cell migration and invasion *in vitro*

**Figure 5:**
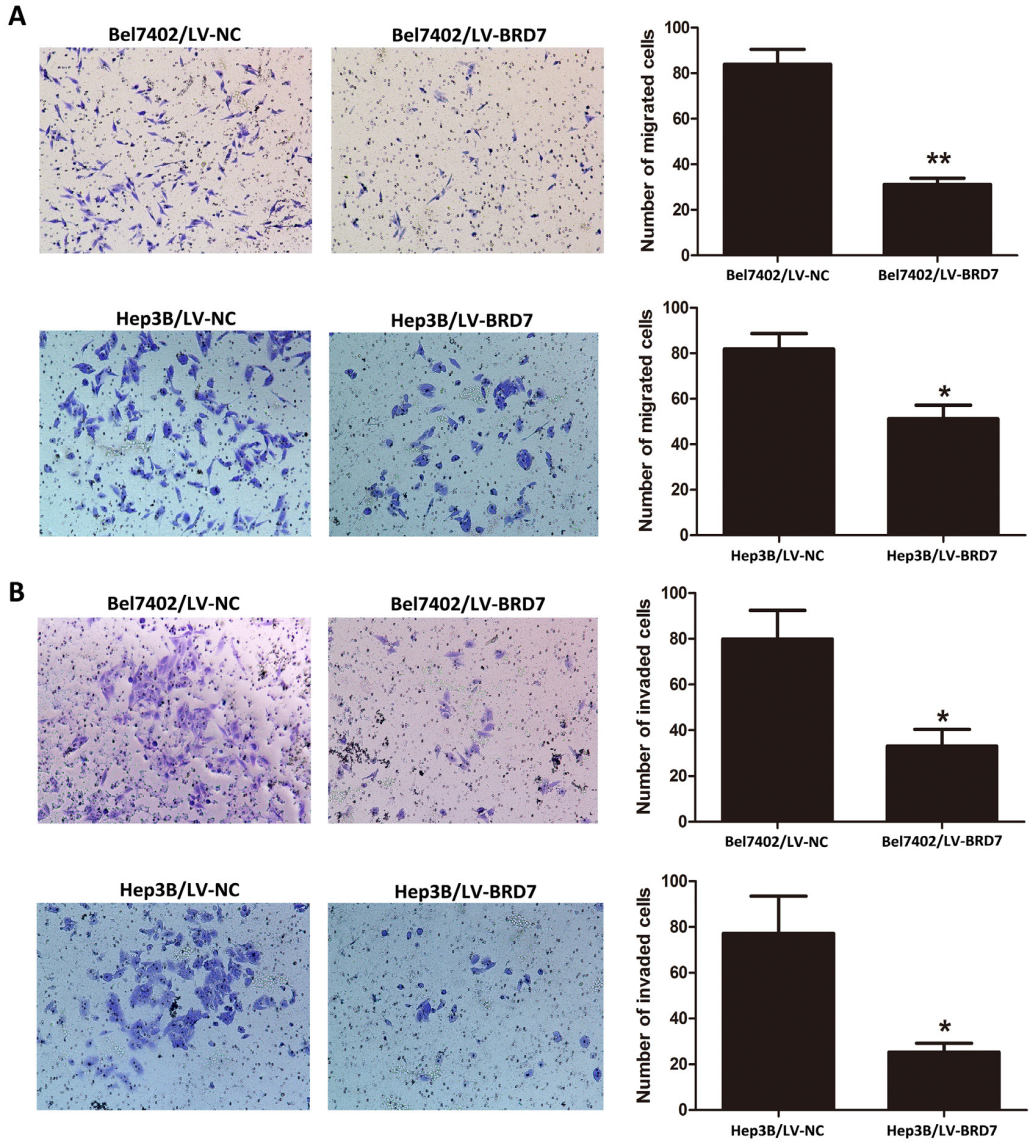
Cell migration and invasion ability of Bel7402 and Hep3B cells transfected with LV-BRD7 and LV-Vector **A.** BRD7 overexpression inhibited the migration ability of Bel7402 and Hep3B cells in a Transwell migration assay. **B.** BRD7 overexpression also significantly attenuated the invasion ability of Bel7402 and Hep3B cells in a Matrigel invasion assay. Images are shown on the left at 100× magnification; the quantification of 6 randomly selected fields is shown on the right. The data shown are expressed as the mean ± SD of three independent experiments. *P*-values were calculated using the independent Student's *t*-test. *, *P* < 0.05 *versus* LV-Vector; **, *P* < 0.01 *versus* LV-Vector.

Due to the significant relationship between decreased BRD7 expression and TNM stage revealed in the correlation analysis, we subsequently performed cell-migration and invasion assays to determine the effects of BRD7 expression on metastasis in HCC. We found that the migration ability of LV-BRD7-transfected Bel7402 and Hep3B cells was significantly reduced compared with LV-Vector-transfected cells (Figure 5A). Consistent with these results, BRD7 also significantly inhibited cell invasion into the lower compartment of a Matrigel-coated chamber (Figure 5B). These results indicated that BRD7 may prevent cell invasion and metastasis in HCC patients.

### BRD7 suppresses HCC tumorigenesis *in vivo*

**Figure 6:**
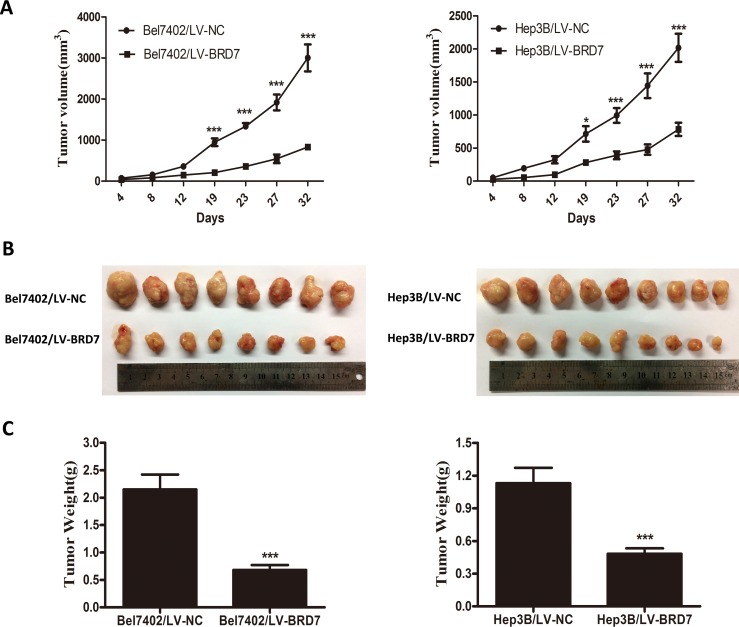
BRD7 suppresses the tumorigenicity of HCC *in vivo* **A.** The tumor growth curves for each group are shown. BRD7 overexpression markedly reduced the tumor growth rate of HCC *in vivo*. **B.** Photographs of dissected tumors from the nude mice. The final tumor volumes were much smaller in the Bel7402/LV-BRD7 and Hep3B/LV-BRD7 transfection groups compared with the Bel7402/LV-NC and Hep3B/LV-NC transfection groups. **C.** The tumor weights of each group are shown. The final tumor weights were lower in the tumors that overexpressed BRD7. The data are presented as the means ± SD. P-values were calculated using the independent Student's *t*-test. *, *P* < 0.05 *versus* LV-Vector; ***, *P* < 0.001 *versus* LV-Vector.

To assess the role of BRD7 in HCC tumorigenesis *in vivo*, we established a xenograft nude mouse model *via* the subcutaneous injection of Bel7402 or Hep3B cells transfected with LV-BRD7 or LV-Vector. Tumor growth was significantly delayed in nude mice that were injected with LV-BRD7-transfected Bel7402 or Hep3B cells compared with mice that were injected with LV-Vector-transfected cells (Figure 6A). At the end of the observation period, the mean tumor volume in the mice that were injected with BRD7 was significantly smaller than that in the mice that were injected with empty vector (831.07 mm^3^ *vs*. 3003.56 mm^3^ for Bel7402, 782.84 mm^3^ *vs*. 2015.89 mm^3^ for Hep3B; Figure 6A and 6B). Furthermore, smaller mean tumor weights were found in the mice that received BRD7-expressing cells compared with those that were injected with empty vector (0.683 g *vs*. 2.150 g for Bel7402, 0.482 g *vs*. 1.131 g for Hep3B; Figure 6C).

## DISCUSSION

Although recent major advances in diagnosis and therapy have increased the likelihood of a cure for early HCC, the long-term prognosis of patients with HCC remains dismal due to rapid tumor progression and tumor relapse [23, 24]. Hepatocarcinogenesis is considered a complex multistep process that involves numerous genetic alterations and signaling pathways, such as p53 mutations and PI3K signaling [7, 25]. These genomic alterations and signaling pathways, which are linked to distinct cellular processes, including metabolism, cell proliferation, apoptosis, migration, invasion and the cell cycle, play important roles in the development of HCC [26]. Achieving accurate diagnosis, precise therapy and a lasting cure for advanced HCC requires deepening our understanding of HCC and identifying specific biomarkers that are involved in these genetic alterations and signaling pathways.

BRD7, a subunit of the PBAF-specific SWI/SNF chromatin-remodeling complex, plays a key role as a transcriptional cofactor in the regulation of several important tumor suppressors and tumor-related signaling pathways [27, 28]. For example, recent work has demonstrated that BRD7 is required for the transcriptional activation of the p53 gene, as well as p53 target genes such as p21 and TIGAR [29]. In addition to its role in the SWI/SNF complex and in promoting p53 function, BRD7 also functions as a direct regulator of PI3K signaling through its interaction with p85α [18]. Accumulating evidence indicates that the down-regulation of the BRD7 gene contributes to the development of multiple types of human cancer [30, 31]. For instance, BRD7 mRNA expression is much higher in normal nasopharyngeal epithelia compared with NPC biopsies and cell lines [32]. Recently, Kaishun Hu *et al*. revealed that the degradation of BRD7 by APC/C^cdh1^ and APC/C^cdc20^ blocks the inhibitory effects of BRD7 on the tumorigenesis of osteosarcoma [19]. To date, however, the biological functions and clinical significance of BRD7 in HCC remain unknown.

In this study, we presented the first evidence that BRD7 expression was significantly down-regulated at both the mRNA and protein level in most primary HCC tumor tissues and in HCC cell lines compared with corresponding noncancerous tissues and normal liver cells. Consistent with these molecular biological findings, our immunohistochemical analysis also revealed that BRD7 expression was lower in HCC tissue samples than that in matched adjacent noncancerous tissues. Decreased BRD7 expression was found to be significantly associated with TNM stage and tumor size. These observations indicated that the loss of BRD7 may promote the development and progression of HCC by facilitating tumor growth, invasion and infiltration. Kaplan-Meier survival analysis revealed a significant correlation between lower BRD7 expression and unfavorable prognosis in HCC patients following radical surgery. Furthermore, a multivariate analysis demonstrated that BRD7 expression was a potential independent prognostic factor for the survival of patients with HCC. Indeed, the tumor suppressor characteristics of BRD7 are well known. A previous report showed that decreased BRD7 expression might play a crucial role in the aggressive behavior of high-grade ovarian tumors and may potentially serve as a prognostic marker for ovarian cancer [22]. Other investigators also found that low/absent BRD7 expression was significantly associated with clinicopathological characteristics and poor clinical outcomes in patients with colorectal cancer [21]. Moreover, lower BRD7 expression was also identified as an indicator of poor prognosis in patients with osteosarcoma [19].

To further investigate the potential mechanism by which BRD7 suppresses the development and progression of HCC, we conducted a series of functional studies by transfecting full-length BRD7 into Bel7402 and Hep3B HCC cells, which exhibit low BRD7 expression. Restoring BRD7 expression significantly suppressed cell proliferation and colony formation in these cells. In parallel experiments, a role for BRD7 in inhibiting tumor growth was also observed in injectable mouse models. BRD7 overexpression also contributed to G0/G1 phase arrest and reduced the percentage of HCC cells in G2/M phase, indicating that BRD7 suppresses HCC tumorigenicity by inducing cell cycle arrest. Consistent with our results, previous observations have also demonstrated that BRD7 can inhibit cell growth through cell cycle arrest. For example, Jie Zhou *et al.* found that BRD7 markedly inhibited NPC cell growth and G1-S cell-cycle progression through the transcriptional regulation of ras/MEK/ERK and Rb/E2F signaling pathway components [32]. A similar observation demonstrated that BRD7 regulated the growth suppression of NPC cells by arresting cells in G1 by the inhibiting both the beta catenin and ERK1/2 pathways [15]. Ming Zhou *et al.* also confirmed that BRD7 with a functional nuclear localization signal (NLS) is critical for the down-regulation of cyclin D1 and E2F3 protein expression, as well as for the inhibition of cell cycle progression from G1 to S phase [33]. Moreover, our study found that BRD7 overexpression suppressed the migration and invasion of Bel7402 and Hep3B cells *in vitro*, which suggests that BRD7 may play a role in tumor metastasis. These findings also explain why decreased BRD7 expression was significantly correlated with TNM stage.

In summary, our study results are the first to show that the bromodomain-containing protein BRD7 is a novel prognostic biomarker for HCC, as evidenced by the down-regulation of BRD7 at both the transcriptional and translational levels in the majority of HCC tissue samples and cell lines tested and by the significant correlation between low BRD7 expression and poor prognosis in HCC patients. Cell culture studies confirmed that BRD7 suppressed HCC tumorigenicity by inhibiting proliferation, colony formation, migration/invasion and by inducing cell cycle arrest in tumor cells. The mouse model experiments further showed that BRD7 overexpression significantly inhibited tumor growth. Taken together, our results suggested that BRD7 serve as a potential tumor suppressor and as a new molecular target for the treatment of HCC, although the precise molecular mechanism by which BRD7 regulates HCC progression remains to be elucidated.

## MATERIALS AND METHODS

### Cell lines

The HCC cell lines Hep3B and HepG2 were purchased from the American Type Culture Collection (ATCC, Manassas, VA, USA). The HCC cell lines Bel7402 and HCCLM6, as well as the normal liver cell line LO2, were obtained from the Committee of Type Culture Collection of the Chinese Academy of Sciences (Shanghai, China). All cells were cultured in RPMI 1640 medium (Invitrogen, Shanghai, China) supplemented with 10% fetal bovine serum (FBS; Gibco, NY, USA) and 100 units/ml penicillin plus 100 μg/ml streptomycin. Cells were incubated at 37°C in a humidified chamber containing 5% CO_2_ in air.

### Patients and tumor tissue samples

A total of 159 human primary HCC tissues were randomly collected from HCC patients who underwent hepatectomy at the Sun Yat-sen University Cancer Center (SYSUCC) between 2004 and 2010. All tissue samples were fixed in 10% formalin and embedded in paraffin, and consecutive 3-μm-thick sections were cut. The diagnosis was histologically confirmed by pathological examination, and none of the patients had received preoperative radiotherapy or chemotherapy. The histological type and clinical stage of HCC were determined according to the WHO classification criteria and the TNM stage established by The American Joint Committee on Cancer (AJCC) [34], respectively. All of the postoperative patients in this study underwent regular follow-up at our outpatient department or at our follow-up center. The follow-up data were obtained every 3 months for the first 2 years, every 6 months from years 3 to years 5, and annually thereafter, or until patient death, whichever occurred first. The follow-up data included clinical and laboratory examinations such as abdominal ultrasonography, liver function tests, serum AFP levels, and chest radiography. OS and RFS were used to measure prognosis in this study. OS was defined as the time from the date of surgery to the date of death or the last follow-up. RFS was defined as the time from the date of surgery to the date of the first detectable recurrence or the final follow-up. In addition, 40 pairs of fresh tumor samples and matched adjacent noncancerous liver tissue samples were collected from HCC patients who underwent hepatectomy at SYSUCC between 2013 and 2014. After surgical resection, the fresh samples were immediately immersed in RNAlater (Ambion; Carlsbad, CA, USA) to prevent RNA degradation, stored at 4°C overnight, and then frozen at −80°C until RNA isolation and protein extraction were performed. This study was approved by the Ethics Committee of Sun Yat-sen University Cancer Center, and written consent was obtained from each patient.

### RNA extraction and real-time quantitative reverse transcriptase polymerase chain reaction (qRT-PCR)

Total RNA was extracted from the 40 paired tissue samples and from the cell lines (LO2, HepG2, Hep3B, Bel7402 and HCCLM6) using TRIzol reagent (Invitrogen, Carlsbad, CA, USA) according to the manufacturer's protocol. The total RNA concentration and purity were measured by absorbency at 260 nm on a NanoDrop spectrophotometer (ND-1000, Thermo Scientific, USA). First-strand cDNA synthesis was performed on 1 μg of total RNA using GoScript™ Reverse Transcriptase (Promega, Beijing, China) according to the manufacturer's recommendations. qRT-PCR analysis was performed with the resulting cDNAs to assess the relative mRNA expression levels of BRD7 and GAPDH (an internal control). The primer sequences used for qRT-PCR were as follows: 5′-TCTCTTGGGTCCCTCATACAG-3′ (forward) and 5′-CACTCAGCAACATCCGTCTT-3′ (reverse) for BRD7; 5′- CTCCTCCTGTTCGACAGTCAGC -3′ (forward) and 5′- CCCAATACGACCAAAT CCGTT -3′ (reverse) for GAPDH. Gene-specific amplification was performed in an ABI Prism 7900HT Sequence Detection System (Life Technologies, Carlsbad, CA, USA) in a final volume of 15 μl SYBR Green Master Mix, which contained the following: 0.5 μl of cDNA (synthesized as described above), 7.5 μl of 2x SYBR Green Master Mix (Invitrogen, USA), 2 μl of each pair of oligonucleotide primers and 5 μl of nuclease-free water. The amplification protocol was as follows: 95°C for 10 min, followed by 40 cycles of 95°C for 30 sec and 60°C for 60 sec. Specificity was identified by melting-curve analysis. The crossing threshold (Ct) value was obtained during the exponential amplification phase using the instrument's software (SDS v.2.3). The relative expression level of BRD7 mRNA was calculated and normalized to that of GAPDH using the comparative threshold cycle (2-ΔΔCT) method. All experiments were performed in triplicate.

### Protein extraction and western blotting

Proteins were extracted from the same fresh tissue samples and cell lines used for RNA extraction. The samples were homogenized in ice-cold RIPA lysis buffer (Beyotime, Shanghai, China), and the lysates were then centrifuged (12,000 rpm) at 4°C for 30 min. The supernatants were harvested, and protein concentrations were measured with a BCA Protein Assay Kit (Bio-Rad; Hercules, CA, USA). After quantification, approximately 30 μg of protein from each sample was separated by 12% SDS-PAGE and transferred onto polyvinylidene fluoride (PVDF) membranes (Millipore, Guangzhou, China). The membranes were blocked for 60 min with 5% non-fat milk and were then incubated with rabbit anti-BRD7 (1:1000; Cell Signaling Technology, Inc, USA) or rabbit anti-GAPDH (1:5000; Proteintech, Chicago, IL, USA) overnight at 4°C. After three 10-min washes in phosphate-buffered saline supplemented with Tween 20 (PBST), the membranes were probed with HRP-conjugated secondary antibody (1:2000; Cell Signaling Technology, Inc, USA) at room temperature for 1 h. After three more washes in PBST, the membranes were developed using an enhanced chemiluminescence detection system (ECL, Cell Signaling Technology, Inc, USA). The intensity of the protein bands on the western blots was measured using ImageJ software (NIH, Bethesda, MD, USA). The target protein levels were normalized to the corresponding GAPDH protein levels.

### Immunohistochemistry and semi-quantitative analysis

The 159 paraffin-embedded HCC tissues were sectioned at a thickness of 3 μm, deparaffinized and rehydrated. After three washes in PBS, antigen retrieval was performed as follows: the sections were boiled in EDTA (1 mM, pH 8.0) in a microwave oven at 100°C for 15 min. Then, the sections were incubated in 0.3% hydrogen peroxide for 10 min at room temperature to block endogenous peroxidase activity, followed by incubation with sheep serum albumin for 30 min to prevent non-specific binding. Then, the sections were incubated with a primary antibody against BRD7 (1:300, Proteintech, Chicago, IL, USA) at 4°C overnight. After the slides were rinsed five times with PBS, they were incubated for 30 min at room temperature with horseradish peroxidase-conjugated secondary antibody as part of an Envision Detection Kit (GK500705, Gene Tech; Shanghai, China). The sections were then washed five more times in PBS. Finally, the sections were incubated with 3,3′-diaminobenzidine (DAB) to visualize the signal and were counter-stained with hematoxylin. As a negative control, adjacent sections were incubated under the same experimental conditions without the primary antibody.

The total BRD7 immunostaining observed on each section was scored as the sum of the staining intensity and the percentage of positively stained tumor cells. Briefly, the intensity was classified as “0” (no staining), “1” (weakly stained), “2” (moderately stained) or “3” (well stained), and the percent positivity was scored as “0” (< 5%, negative), “1” (5%-25%, sporadic), “2” (25%-50%, focal), or “3” (≥50%, diffuse). The score was calculated independently by two experienced pathologists who were blinded to the clinical outcomes; any discrepancies in scoring were adjudicated. The values of the intensity and the percent positivity were multiplied to obtain the final immunostaining scores for BRD7 (scores ranged from 0 to 9). BRD7 expression levels were defined as follows: “-” (negative, score 0-1), “+” (weakly positive, score 2-3), “++” (positive, score 4-6), “+++” (strongly positive, score 9). Based on the BRD7 expression levels, the HCC tissue samples were divided into a high BRD7 expression group (BRD7++ or BRD7+++) and a low BRD7 expression group (BRD7- or BRD7+).

### Generation of stable BRD7-overexpressing cell lines

Recombinant lentiviruses that overexpressed BRD7 (designated as LV-BRD7) or empty control vector (designated as LV-Vector) were obtained from GenePharma (Shanghai, China). Bel7402 and Hep3B cells were each cultured in 6-well plates until they reached 70% confluence. The lentiviruses were then added to the cell culture medium along with 5 mg/ml polybrene (Sigma-Aldrich, St. Louis, MO, USA) for infection. After 48 h, 5 μg/ml puromycin was added to the cells to generate stable transformants. Finally, puromycin-resistant cells were selected and cultured for further analysis. The stable cell lines were designated as Bel7402/LV-BRD7, Bel7402/LV-NC, Hep3B/LV-BRD7, or Hep3B/LV-NC.

### Colony formation assay

For the colony formation assay, cells that were transfected with LV-BRD7 or LV-Vector were plated in 6-well plates at a density of 1,000 cells per well. After incubation for 10 days at 37°C in a humidified chamber containing 5% CO_2_, the cells were washed with normal saline and fixed in 75% ethanol. The surviving colonies (> 50 cells) were counted after being stained with 0.5% crystal violet for approximately 30 min. The colony-forming efficiency (CFE %) was calculated as follows: CFE % = (colony number/plated cell number) × 100. This experiment was performed in triplicate.

### Proliferation assay

For the cell proliferation assay, the growth rate of the infected cells was measured using an MTS cell proliferation assay. Briefly, the cells were plated in 96-well plates at a density of 1,500 cells per well, and growth was evaluated using an MTS cell proliferation kit (Promega, Madison, WI, USA) according to the manufacturer's instructions. Three independent experiments were performed.

### Cell cycle assay

The cell cycle analysis was performed *via* propidium iodide (PI) flow cytometry. Bel7402 and Hep3B cells were routinely collected and washed three times in ice-cold PBS 72 h after transfection. Then, the cells were fixed in ice-cold 75% ethanol at −20°C overnight. After being rinsed twice in PBS, the cells were resuspended in PBS and incubated with RNase at 37°C for 30 min. The cells were subsequently stained with PI (Bestbio, Shanghai, China) for 60 min at 4°C in the dark. The cell cycle distribution was analyzed *via* flow cytometry (Beckman, Fullerton, USA). All experiments were performed in triplicate.

### Cell-migration and invasion assays

The cell-migration and invasion assays were performed using a 24-well Transwell chamber system that consisted of polycarbonate membrane inserts with 8-μm pores (Corning Incorporated, Corning, NY, USA). For the cell-migration assay, 1 × 10^5^ cells were plated in the upper chamber (without Matrigel). For the cell-invasion assay, 2 × 10^5^ cells were plated in the upper chamber, which was pre-coated with a thin layer of 0.5 mg/ml Matrigel (BD, Franklin Lakes, NJ, USA). For both assays, 600 μl of RPMI 1640 medium supplemented with 10% FBS was added to the lower chamber, while 200 μl of RPMI 1640 medium without FBS was placed in the upper chamber. The migration and invasion assays were performed after the cells were incubated at 37°C for 24 or 48 h, respectively. The cells that remained in the upper chamber were removed with cotton-tipped swabs. The cells that had migrated or invaded to the lower chamber were then fixed in 75% ethanol for 30 min and stained with 0.5% crystal violet for 60 min. The migration and invasion efficiencies were evaluated by counting the stained cells on an inverted microscope (6 fields were randomly selected per membrane). Both assays were conducted 48 h after the Bel7402 and Hep3B cells were infected, and each experiment was performed in triplicate.

### Tumorigenicity assays in nude mice

Female BALB/c nude mice (4 weeks old) were purchased from the Medical Experimental Animal Center of Guangdong Province. The mice were randomly assigned to 4 groups of 10 mice, which were subcutaneously injected with a total of 4×10^6^ Bel7402/LV-BRD7, Bel7402/LV-NC, Hep3B/LV-BRD7 or Hep3B/LV-NC cells suspended in 100 μl of PBS supplemented with 30% Matrigel Basement Membrane Matrix (BD). The tumor size was estimated every 4 days by measuring the length and width of the tumor with a sliding caliper. The tumor volume was calculated using the formula V = 1/2 (length×width^2^). All mice were euthanized approximately 5 weeks after inoculation. The tumors were harvested, photographed and weighed. All animal experiments were conducted according to the Guide for the Care and Use of Laboratory Animals (NIH publication Nos. 80-23, revised 1996) and the institutional ethical guidelines for animal experiments.

### Statistical analysis

All statistical analyses were performed using the Statistical Package for the Social Sciences version 20.0 software (SPSS Inc., Chicago, IL, USA). The Mann-Whitney *U*-test was used to compare BRD7 mRNA and protein expression between the tumor samples and the matched adjacent noncancerous liver tissue samples. Student's *t*-test, the Pearson *χ*^2^ test and Fisher's exact test were used to determine the correlation between BRD7 expression and the clinical variables of the HCC patients. OS and RFS curves were calculated using the Kaplan-Meier method and were analyzed using the log-rank test. Univariate and multivariate regression analyses were performed with a Cox proportional-hazard model to investigate the effects of BRD7 expression and HCC clinical characteristics on survival. Only those factors that were considered statistically significant in the univariate analysis were included in the multivariate Cox proportional hazards model to analyze the effects of the covariates. The results are shown as the mean ± SD and were analyzed using Student's *t*-test. All tests were two-sided, and *P* < 0.05 was considered statistically significant.

## References

[1] Jemal A, Bray F, Center MM, Ferlay J, Ward E, Forman D (2011). Global cancer statistics. CA Cancer J Clin.

[2] El-Serag HB (2012). Epidemiology of viral hepatitis and hepatocellular carcinoma. Gastroenterology.

[3] Ferlay J, Shin HR, Bray F, Forman D, Mathers C, Parkin DM (2010). Estimates of worldwide burden of cancer in 2008: GLOBOCAN 2008. Int J Cancer.

[4] Ulahannan SV, Duffy AG, McNeel TS, Kish JK, Dickie LA, Rahma OE, McGlynn KA, Greten TF, Altekruse SF (2014). Earlier presentation and application of curative treatments in hepatocellular carcinoma. Hepatology.

[5] Llovet JM, Ricci S, Mazzaferro V, Hilgard P, Gane E, Blanc JF, de Oliveira AC, Santoro A, Raoul JL, Forner A, Schwartz M, Porta C, Zeuzem S (2008). Sorafenib in Advanced Hepatocellular. N Engl J Med.

[6] El-Serag HB (2011). Hepatocellular carcinoma. N Engl J Med.

[7] Farazi PA, DePinho RA (2006). Hepatocellular carcinoma pathogenesis: from genes to environment. Nat Rev Cancer.

[8] Villanueva A, Newell P, Chiang DY, Friedman SL, Llovet JM (2007). Genomics and signaling pathways in hepatocellular carcinoma. Semin Liver Dis.

[9] Zhu AX, Duda DG, Sahani DV, Jain RK (2011). HCC and angiogenesis: possible targets and future directions. Nat Rev Clin Oncol.

[10] Cuppen E, van Ham M, Pepers B, Wieringa B, Hendriks W (1999). Identification and molecular characterization of BP75, a novel bromodomain-containing protein. FEBS Lett.

[11] Jeanmougin F, Wurtz JM, Le Douarin B, Chambon P, Losson R (1997). The bromodomain revisited. Trends Biochem Sci.

[12] Dyson MH, Rose S, Mahadevan LC (2001). Acetyllysine-binding and function of bromodomain-containing proteins in chromatin. Front Biosci.

[13] Kaeser MD, Aslanian A, Dong MQ, Yates JR, Emerson BM (2008). BRD7, a novel PBAF-specific SWI/SNF subunit, is required for target gene activation and repression in embryonic stem cells. J Biol Chem.

[14] Peng C, Liang SP, Tan C, Lv HB, Huang H, Zhou M, Wang R, Li XL, Li GY (2003). Researching a novel NPC-related candidate suppressor gene BRD7 by two-dimensional gel electrophoresis and MALDI-TOF-MS [Article in Chinese]. Sheng Wu Hua Xue Yu Sheng Wu Wu Li Xue Bao (Shanghai).

[15] Peng C, Liu HY, Zhou M, Zhang LM, Li XL, Shen SR, Li GY (2007). BRD7 suppresses the growth of Nasopharyngeal Carcinoma cells (HNE1) through negatively regulating beta-catenin and ERK pathways. Mol Cell Biochem.

[16] Drost J, Mantovani F, Tocco F, Elkon R, Comel A, Holstege H, Kerkhoven R, Jonkers J, Voorhoeve PM, Agami R, Del Sal G (2010). BRD7 is a candidate tumour suppressor gene required for p53 function. Nat Cell Biol.

[17] Harte MT, O'Brien GJ, Ryan NM, Gorski JJ, Savage KI, Crawford NT, Mullan PB, Harkin DP (2010). BRD7, a subunit of SWI/SNF complexes, binds directly to BRCA1 and regulates BRCA1-dependent transcription. Cancer Res.

[18] Chiu YH, Lee JY, Cantley LC (2014). BRD7, a tumor suppressor, interacts with p85α and regulates PI3K activity. Mol Cell.

[19] Hu K, Liao D, Wu W, Han AJ, Shi HJ, Wang F, Wang X, Zhong L, Duan T, Wu Y, Cao J, Tang J, Sang Y (2014). Targeting the anaphase-promoting complex/cyclosome (APC/C)- bromodomain containing 7 (BRD7) pathway for human osteosarcoma. Oncotarget.

[20] Kikuchi M, Okumura F, Tsukiyama T, Watanabe M, Miyajima N, Tanaka J, Imamura M, Hatakeyama S (2009). TRIM24 mediates ligand-dependent activation of androgen receptor and is repressed by a bromodomain-containing protein, BRD7, in prostate cancer cells. Biochim Biophys Acta.

[21] Wu WJ, Hu KS, Chen DL, Zeng ZL, Luo HY, Wang F, Wang DS, Wang ZQ, He F, Xu RH (2013). Prognostic relevance of BRD7 expression in colorectal carcinoma. Eur J Clin Invest.

[22] Park YA, Lee JW, Kim HS, Lee YY, Kim TJ, Choi CH, Choi JJ, Jeon HK, Cho YJ, Ryu JY, Kim BG, Bae DS (2014). Tumor suppressive effects of bromodomain-containing protein 7 (BRD7) in epithelial ovarian carcinoma. Clin Cancer Res.

[23] Maluccio M, Covey A (2012). Recent progress in understanding, diagnosing, and treating hepatocellular carcinoma. CA Cancer J Clin.

[24] Bruix J, Gores GJ, Mazzaferro V (2014). Hepatocellular carcinoma: clinical frontiers and perspectives. Gut.

[25] Janku F, Kaseb AO, Tsimberidou AM, Wolff RA, Kurzrock R (2014). Identification of novel therapeutic targets in the PI3K/AKT/mTOR pathway in hepatocellular carcinoma using targeted next generation sequencing. Oncotarget.

[26] Garraway LA, Lander ES (2013). Lessons from the cancer genome. Cell.

[27] Mantovani F, Drost J, Voorhoeve PM, Del Sal G, Agami R (2010). Gene regulation and tumor suppression by the bromodomain-containing protein BRD7. Cell Cycle.

[28] Liu H, Zhou M, Luo X, Zhang L, Niu Z, Peng C, Ma J, Peng S, Zhou H, Xiang B, Li X, Li S, He J (2008). Transcriptional regulation of BRD7 expression by Sp1 and c-Myc. BMC Mol Biol.

[29] urrows AE, Smogorzewska A, Elledge SJ (2010). Polybromo-associated BRG1-associated factor components BRD7 and BAF180 are critical regulators of p53 required for induction of replicative senescence. Proc Natl Acad Sci U S A.

[30] Sokolenko AP, Preobrazhenskaya EV, Aleksakhina SN, Iyevleva AG, Mitiushkina NV, Zaitseva OA, Yatsuk OS, Tiurin VI, Strelkova TN, Togo AV, Imyanitov EN (2015). Candidate gene analysis of BRCA1/2 mutation-negative high-risk Russian breast cancer patients. Cancer Lett.

[31] Tang H, Wang Z, Liu Q, Liu X, Wu M, Li G (2014). Disturbing miR-182 and -381 inhibits BRD7 transcription and glioma growth by directly targeting LRRC4. PLoS One.

[32] Zhou J, Ma J, Zhang BC, Li XL, Shen SR, Zhu SG, Xiong W, Liu HY, Huang H, Zhou M, Li GY (2004). BRD7, a novel bromodomain gene, inhibits G1-S progression by transcriptionally regulating some important molecules involved in ras/MEK/ERK and Rb/E2F pathways. J Cell Physiol.

[33] Zhou M, Liu H, Xu X, Zhou H, Li X, Peng C, Shen S, Xiong W, Ma J, Zeng Z, Fang S, Nie X, Yang Y (2006). Identification of nuclear localization signal that governs nuclear import of BRD7 and its essential roles in inhibiting cell cycle progression. J Cell Biochem.

[34] Edge SB, Compton CC (2010). The American Joint Committee on Cancer: the 7th edition of the AJCC cancer staging manual and the future of TNM. Ann Surg Oncol.

